# ABC Dementia Scale Classifies Alzheimer’s Disease Patients into Subgroups Characterized by Activities of Daily Living, Behavioral and Psychological Symptoms of Dementia, and Cognitive Function

**DOI:** 10.3233/JAD-190767

**Published:** 2020-01-07

**Authors:** Kenji Wada-Isoe, Takashi Kikuchi, Yumi Umeda-Kameyama, Takahiro Mori, Masahiro Akishita, Yu Nakamura, on behalf of the ABC Dementia Scale Research Group

**Affiliations:** a Department of Dementia Research, Kawasaki Medical School, Kita-ku, Okayama, Japan; b Translational Research Informatics Center for Medical Innovation, Foundation for Biomedical Research and Innovation at Kobe, Chuo-ku Kobe, Hyogo, Japan; c Department of Geriatric Medicine, Graduate School of Medicine, The University of Tokyo, Bunkyo-ku, Tokyo, Japan; d Department of Neuropsychiatry, Faculty of Medicine, Kagawa University, Miki-cho, Kita-gun, Kagawa, Japan

**Keywords:** ABC dementia scale, activities of daily living, Alzheimer’s disease, cognitive function, cluster analysis, psychological symptoms of dementia

## Abstract

The course of Alzheimer’s disease (AD) varies between individuals, and the relationship between cognitive and functional decline and the deterioration of behavioral and psychological symptoms of dementia (BPSD) is still poorly understood. Until recently, it was challenging to monitor subsequent changes in these symptoms because there was no single composite scale available that could simultaneously evaluate activities of daily living (ADL), BPSD, and cognitive function (CF) states. The present authors developed a new, brief assessment scale, the “ABC Dementia Scale” (ABC-DS), which is based on item response theory and facilitates concurrent measurement of ADL, BPSD, and CF states. We previously presented the reliability, construct validity, concurrent validity, and responsiveness of the ABC-DS. We obtained the evidence through three clinical trials featuring 1,400 subjects in total. In the present study, we performed a secondary analysis of the data obtained in the previous study. We conducted hierarchical cluster analyses that allowed us to classify 197 AD patients in terms of similarities regarding ADL, BPSD, and CF domain scores, as measured by the ABC-DS. Consequently, the scale identified subgroups of patients with global clinical dementia ratings of 1, 2, and 3. Considering our results in conjunction with the clinical experiences of the AD expert among the present authors regarding longitudinal changes in ADL, BPSD, and CF, we were able to propose potential progression pathways of AD in the form of a hypothetical roadmap.

## INTRODUCTION

Patients with Alzheimer’s disease (AD) are very heterogeneous regarding their profiles of activities of daily living (ADL), their behavioral and psychological symptoms of dementia (BPSD), and their cognitive function (CF).

Previous researchers classified subtypes of AD using various methods such as clinical subtypes defined by diagnostic criteria [1–5]; atrophy subtypes assessed using magnetic resonance imaging [6–11]; etiological subtypes or molecular subtypes characterized by pathological levels of cerebrospinal fluid Aβ_1 - 42_, total tau, and phosphorylated tau [12, 13]; and subtypes based on statistical analyses, such as latent class analysis of Mini-Mental Examination (MMSE) scores [14] and principal component analysis and cluster analysis of the distribution and abundance of senile plaques and neurofibrillary tangles in the brain [15, 16]. Staging using assessment scales is another method of understanding AD pathology [17, 18]. Identifying subtypes through these methods may successfully uncover some aspects of AD and help categorize their severity. However, such approaches do not provide enough information regarding the heterogeneity of changes in ADL, BPSD, and CF as the disease progresses because these approaches do not focus on the combined changes in ADL, BPSD, and CF.

To effectively treat AD, clinicians must understand the precise progression pathways of the disease, considering changes in ADL, BPSD, and CF. For this purpose, we need a conceptual roadmap that indicates the paths of the changes in ADL, BPSD, and CF that patients are likely to experience during disease progression. By presenting these pathways, such a roadmap would contain much more information than the ratings from the currently available assessment scales for dementia.

For the present study, we used PubMed to find numerous studies that examined longitudinal changes in AD symptoms [19–25]; however, we did not find any studies that concurrently monitored longitudinal changes in ADL, BPSD, and CF. The majority of the reviews we found only followed the progression of ADL and CF. Such a research design was probably unavoidable for these previous studies because the researchers needed to use several assessment scales, given the lack of a single tool to make a quick evaluation of ADL, BPSD, and CF together.

We have previously validated a brief assessment scale—the “ABC Dementia Scale” (ABC-DS)—which can concurrently evaluate ADL, BPSD, and CF in ten minutes on average [26–28]. We developed a novel algorithm for this scale called the three-dimensional distance (TDD), for estimating the overall severity of each state while taking into account the fact that ADL, BPSD, and CF levels should explain a patient’s disease condition.

In a recent study, we used hierarchical cluster analysis classify individuals with a global Clinical Dementia Rating (CDR) [17] of 0.5 into three subgroups [29]. In the present study, we conducted hierarchical cluster analysis to classify patients with a CDR of 1, 2, and 3, and to identify several subgroups. We also proposed a hypothetical roadmap of the disease’s progression based on the assumption that ADL, CF, and TDD scores decrease as the disease progresses. We tried to link potential paths on the roadmap and indicated the likely changes in ADL, BPSD, and CF scores that AD patients could experience. In this paper, we also discuss the progression paths featured on this map by referring to personal clinical experiences and the findings of existing studies.

## MATERIALS AND METHODS

### Patients

This study involves a secondary analysis of data obtained in the TRIAD1412 clinical trial [27, 28]. For TRIAD1412, we selected 312 patients in Japan, including 126 (40.4%) men and 186 (59.6%) women. The mean (standard deviation) age of the patients was 80.6 (±7.1) years, and the mean duration of education was 11.1 (±2.6) years. The inclusion criteria were based on the following diagnosis criteria: 1) AD (based on the Diagnostic and Statistical Manual of Mental Disorders, Fourth Edition, Text Revision criteria [2]); 2) probable AD (based on the requirements of the National Institute on Aging-Alzheimer’s Association [NIA-AA] workgroups, or the National Institute of Neurological and Communicative Disorders and Stroke and the Alzheimer’s Disease and Related Disorders Association [4]); or 3) mild cognitive impairment (MCI; based on the International Working group on MCI criteria [30] or NIA-AA diagnostic criteria [31]).

To determine the severity of AD, trained clinical psychologists rated the 312 patients in terms of CDR. The numbers of patients with a CDR score of 0, 0.5, 1, 2, and 3 were consequently determined to be 4, 110, 99, 65, and 33, respectively; one subject was unavailable for rating. The clinical psychologists used the Japanese version of the CDR and participated in a training seminar to ensure the standardization of testing. For the secondary analysis in this article, we extracted patient data with CDR of 1, 2, and 3; the total sample size was 197 out of 312.

We conducted the TRIAD1412 study following the requirements of the World Medical Association’s Declaration of Helsinki (1964) and its amendments and subsequent clarifications [36]. The institutional review board at Kagawa University approved the study protocol, and all caregivers and participants provided written informed consent. We registered the clinical trial with the University Hospital Medical Information Network (http://www.umin.ac.jp/, No. UMIN000021134).

### ABC-DS

We developed the assessment scale and used the item response theory to examine the quality of the questions. We repeated this process twice (TRIAD1402, parts I and II) and obtained a candidate version for a confirmation study. We validated the candidate by statistical approaches in TRIAD1412 [37]. Finally, we measured the responsiveness of the scale and compared it with other standard scales in TRIAD1412 and TRIAD 1710. The sample sizes of TRIAD1402, TRIAD1412, and TRIAD1710 were 972, 312, and 104, respectively. The TRIAD1412 and TRIAD 1710 studies were conducted to observe the natural history of patients and to estimate the drug effect when changing the treatment regimen, respectively.

The ABC-DS is a composite scale to evaluate the levels of ADL, BPSD, and CF. The scale comprises 13 questions (Q1–13), and each item (question) is answered using a nine-point level, ranging from 1 to 9, or from worst to best. Evaluators interview caregivers regarding recent episodes within one month concerning their patients and choose the most suitable score for each item. No specialized training is required to perform the interviews, and the evaluation time was 10 minutes on average for TRIAD1412 (data not shown).

We originally developed this scale for clinical trials so that we could compare the effects of different treatments. To measure the overall severity of dementia and the states of ADL, BPSD, and CF, we extensively revised the questionnaire descriptions by inspecting the quality of the items. We performed this revision by investigating item response category characteristic curves, following the item response theory [32]. We statistically linked the nine levels at each item with the standardized severity in the Z-scores of ADL, BPSD, or CF by the theory. We then validated the reliability, construct validity, concurrent validity, and responsiveness and also confirmed that the ABC-DS was composed of three domains by confirmatory factor analysis [27]. We completed the development through three clinical trials, TRIAD1402, TRIAD1412, TRIAD1710, comprising 1,388 subjects in total.

We can use the ABC-DS to evaluate the effectiveness of medical interventions by comparing patients’ scores before and after the intervention. There are four approaches for such an evaluation: count per item, domain score, the sum of the 13 items (total score), and TDD score. The domain scores for ADL, BPSD, and CF are calculated by summing the following item scores: “Q1 + Q2 + Q3 + Q4 + Q11 + Q12,” “Q7 + Q8 + Q9,” and “Q5 + Q6 + Q10 + Q13,” respectively. We estimated overall severity using total score or TDD score defined by

$$\sqrt{\mathrm{ADL}\;{\mathrm{score}}^{2}+\mathrm{BPSD}\;{\mathrm{score}}^{2}+\mathrm{CF}\;{\mathrm{score}}^{2}}$$

Although the total score can detect changes in the overall severity, it has a disadvantage; if a patient’s score on the ADL domain increases by 2 points between assessments but the score on the CF domain decreases by 2 points, after adding the two scores to obtain the total score, these changes cancel out to zero. Thus, pathological changes would remain undetected between two measurements. However, TDD can identify the difference in the score. Therefore, we used TDD scores to estimate the overall severity in this study.

Our previous research on the concurrent validity of ABC-DS with standard assessment scales [26] found that the correlation was 0.67, –0.64, 0.70, and –0.83 between ABC-DS scores for ADL, BPSD, CF, and TDD and 1) the corresponding scales in the Disability Assessment for Dementia (DAD), 2) the Neuropsychiatric Inventory (NPI), 3) the MMSE, and 4) Clinical Dementia Rating sum of boxes (CDR-SOB), respectively. Also, the intraclass correlation coefficient of the TDD score was 0.96, which was determined by evaluating scores measured at baseline, and those taken one week later [27]. We measured the changes in scores for the ABC-DS, DAD, NPI, MMSE, and CDR-SOB, and observed the natural history of the patients over 12 weeks; their medical treatments were unchanged during this period [27]. We showed box plots for TDD score and domain scores of ADL, BPSD, and CF at baseline per CDR 0 and 0.5, 1, 2, and 3.

Researchers can download the ABC-DS questionnaire in English, French, Chinese, and Korean from the following site under the terms and conditions specified: https://eprovide.mapi-trust.org/instruments/abc-dementia-scale. Any amendment in the ABC-DS questionnaire is not allowed. As TDD is a patent-protected technique, approval is required from the owner (please contact: E-mail: abc_scale@tri-kobe.org) for any commercial use and clinical trials.

### Statistical methods

We calculated 95% confidence intervals for the changes in scores between baseline and 12-week measurements. We also calculated the coefficient of variation (CV: the standard deviation divided by the mean) to evaluate the precision and repeatability of the repeated measures.

Using the three-domain scores (ADL, BPSD, and CF) of the ABC-DS, we performed hierarchical cluster analysis and used three clustering options “complete,” “average,” and “Ward” methods in hclust [33]. Because these algorithms returned very similar results, only results from the Ward method are reported. We described the annotated R code in the Supplementary Material. We achieved this analysis among individuals with CDR scores of 1, 2, and 3, respectively, at baseline (baseline CDR), so that we would classify individuals with similar score patterns in the same branch or subgroup. We discussed the unique nature of each subgroup by comparing the median values of ADL, BPSD, CF domain, and TDD scores. We presented the results in cluster dendrograms, with each cluster regarded as a “branch” of a “tree:” each branch was distinct from the other branches, and the individuals (often called “leaves”) within each branch had broadly similar ABC-DS profiles to each other. We also calculated the basic statistics per baseline CDR for ABC-DS domain scores, the TDD score, and CDR-SOB, including the minimum, first quarter, median, mean, third quarter, and maximum. We conducted these analyses using the statistical analysis software R×64 3.5.2 [34]; the package and application names were *stats* and *hclust* (https://www.rdocumentation.org/packages/fastcluster/versions/1.1.25/topics/ hclust), respectively, for hierarchical cluster analysis.

## RESULTS

### Box plots of ABC-DS scores per baseline CDR

**Fig.1:**
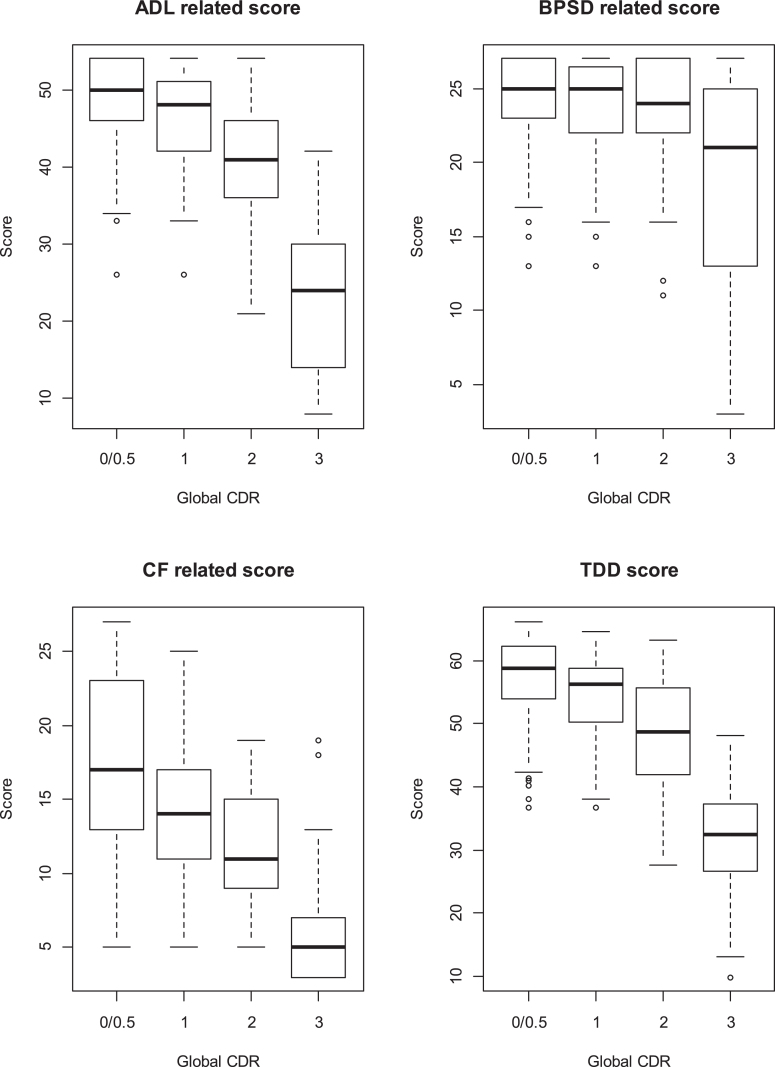
Box plots for ADL, BPSD, and CF domains scores, and TDD scores of ABC-DS that we measured at baseline in TRIAD1412 study. We showed the plots per Global CDR at baseline.

Figure 1 shows that BPSD ratings remained stable through CDR 0–2, unlike ADL and CF. TDD score was, then, primarily driven by a decline in cognitive and functional abilities and declined monotonically as CDR deteriorated. CF score did not hit a floor at the early stages of AD, although there is variability of the score at CDR 0/0.5; CF rapidly declined at CDR 3.

### Changes in scores over 12 weeks

We compared the ABC-DS with other accessible assessment scales (DAD, NPI, MMSE, and CDR-SOB) in terms of 12-week responsiveness (Table 1). If the baseline CDR was 3, all of the scales had 95% confidence intervals that included zero and thus failed to detect the differences. In contrast, for individuals with a baseline CDR of 0 and 0.5, 1, or 2, the TDD score of the ABC-DS revealed changes over the study period because the confidence intervals did not include zero; however, the total ABC-DS score only revealed changes for the group with a baseline CDR of 0 and 0.5 (0/0.5). The MMSE only detected differences among individuals with a baseline CDR of 1, while CDR-SOB did so for baseline CDR scores of 1 and 2; both MMSE and CDR-SOB failed to detect differences among individuals with baseline CDRs of 0/0.5 Also, the absolute values of CV for TDD score, MMSE, and CDR-SOB for individuals with a baseline CDR of 0/0.5 were 3.2, 81.8, and 17.0, respectively, which showed that the TDD score had better precision and replication of the repeated measurements than did MMSE and CDR-SOB.

**Table 1:** 95% confidence intervals for the differences in scores over 12 weeks, during which medical treatment remained unchanged

	Global CDR score at baseline							
	CDR 3		CDR 2		CDR 1		CDR 0.5/0	
Number	33		65		99		114*	
M: F (%)	42.4 : 57.6		33.8 : 66.2		38.8 : 61.2		45.6 : 54.4	
Age	79.5 ∓ 9.0		82.7 ∓ 7.1		81.3 ∓ 6.2		79.1 ∓ 6.9	
Education	10.8 ∓ 2.1		10.1 ∓ 2.3		11.5 ∓ 2.8		11.7 ∓ 2.6	
95% CI	Lower	Upper	Lower	Upper	Lower	Upper	Lower	Upper
ADL	−1.87	0.21	−2.83	−0.27	−2.11	−0.31	−1.21	0.01
BPSD	−2.51	1.40	−0.24	1.64	−0.06	1.02	−0.60	0.11
CF	−0.93	1.26	−2.58	−0.12	−1.82	0.01	−1.56	−0.23
Toatal	−4.18	1.73	−4.55	0.15	−3.09	0.01	−2.81	−0.67
TDD	−2.52	0.83	−2.70	−0.04	−2.03	−0.20	−1.55	−0.31
DAD	−24.88	5.12	−6.68	−1.25	−9.42	−3.78	−5.58	−0.27
NPI	−8.83	0.72	−2.68	4.28	−1.79	2.32	−0.79	1.91
MMSE	−0.29	0.63	−1.46	0.16	−1.31	−0.10	−0.58	0.51
CDR-SOB	−0.03	0.81	0.33	1.27	0.13	0.71	−0.15	0.27

Number, number of subjects; M:F, Male (%): Female (%); Age and Education, mean ∓ standard deviation; ADL, ABC Dementia Scale (ABC-DS) score for activities of daily living (Domain A); BPSD, ABC-DS score for behavioral and psychological symptoms of dementia (Domain B); CDR, Clinical Dementia Rating; CDR, Clinical Dementia Rating sum of boxes; CF, ABC-DS score for cognitive function (Domain C); DAD, Disability Assessment for Dementia; MMSE, Mini-Mental State Examination; NPI, Neuropsychiatric Inventory; TDD, ABC-DS score for three-dimensional distance; Total, the total score for the 13 items of the ABC-DS. Lower: the lower band of 95% confidence interval for the differences in scores. Upper: the upper band of 95% confidence interval for the differences in scores. *Four of 114 subjects had CDR of 0.

### Hierarchical cluster analyses

We cut the cluster dendrogram “tree” at the first generation (branch) and classified the patients with baseline CDR scores of 1, 2, and 3 into four, five, and three subgroups, respectively (Supplementary Figures 1–3). We then obtained the basic statistics per each baseline CDR score for ABC-DS domain scores, TDD score, and CDR-SOB score (Tables 2–5). There were similarities in the ABC-DS’ scores for ADL, BPSD, CF, and TDD between the subgroups of C1.2, C2.1, and C2.2; between C1.4 and C2.3; and between C2.5 and C3.1. Consequently, we grouped C1.2, C2.1, and C2.2 into GI; C1.4 and C2.3 into GII; and C2.5 and C3.1 into GIII.

**Table 2:** ABC-DS and CDR-SOB scores for individuals with a baseline CDR score of 1

Group		ADL	BPSD	CF	TDD	CDR-SOB
C 1.1	Min.	40.0	20.0	19.0	51.6	4.5
*n* = 27	1st Qu.	48.0	24.0	22.0	58.8	5.0
	**Median**	**50.0**	**26.0**	**25.0**	**61.1**	**5.0**
	Mean	49.7	25.2	24.6	61.0	5.9
	3rd Qu.	52.0	27.0	26.5	62.9	7.0
	Max.	54.0	27.0	32.0	68.3	9.0
C 1.2	Min.	37.0	19.0	10.0	48.4	4.0
*n* = 41	1st Qu.	48.0	24.0	16.0	56.0	6.0
	**Median**	**50.0**	**25.0**	**17.0**	**58.5**	**7.0**
	Mean	49.1	24.9	17.7	58.0	6.9
	3rd Qu.	52.0	27.0	19.0	60.1	8.0
	Max.	54.0	27.0	28.0	64.1	10.0
C 1.3	Min.	35.0	15.0	11.0	41.4	4.5
*n* = 11	1st Qu.	41.0	16.5	14.0	48.6	5.3
	**Median**	**43.0**	**18.0**	**16.0**	**50.6**	**6.0**
	Mean	43.7	18.9	16.3	50.6	6.4
	3rd Qu.	47.5	21.5	16.5	53.1	7.5
	Max.	50.0	25.0	28.0	59.2	10.0
C 1.4	Min.	26.0	13.0	7.0	38.0	4.0
*n* = 20	1st Qu.	35.0	21.0	9.8	42.6	6.0
	**Median**	**38.0**	**22.5**	**12.5**	**46.0**	**7.8**
	Mean	37.8	22.3	12.5	45.8	7.4
	3rd Qu.	40.3	25.0	14.0	49.4	9.0
	Max.	44.0	27.0	20.0	52.1	10.0

1st Qu., first quarter; 3rd Qu., third quarter; ADL, ABC Dementia Scale (ABC-DS) score for activities of daily living (Domain A); BPSD, ABC-DS score for behavioral and psychological symptoms of dementia (Domain B); CDR-SOB, Clinical Dementia Rating sum of boxes; CF, ABC-DS score for cognitive function (Domain C); Max., maximum; Min., minimum; TDD, ABC-DS score for three-dimensional distance.

**Table 3:** ABC-DS and CDR-SOB scores for individuals with a baseline CDR score of 2

Group		ADL	BPSD	CF	TDD	CDR-SOB
C2.1	Min.	42.0	22.0	20.0	54.6	9.0
*n* = 9	1st Qu.	46.0	25.0	20.0	57.0	10.0
	**Median**	**48.0**	**26.0**	**22.0**	**58.8**	**11.0**
	Mean	47.4	25.7	22.7	58.6	10.7
	3rd Qu.	49.0	27.0	24.0	59.8	11.0
	Max.	54.0	27.0	26.0	63.6	13.0
C2.2	Min.	36.0	21.0	12.0	46.1	9.0
*n* = 23	1st Qu.	42.5	24.0	14.0	51.9	10.0
	**Median**	**46.0**	**25.0**	**16.0**	**54.7**	**10.5**
	Mean	46.0	25.1	16.3	55.0	10.7
	3rd Qu.	50.0	27.0	18.0	58.3	11.0
	Max.	54.0	27.0	22.0	64.3	14.0
C2.3	Min.	28.0	12.0	7.0	33.0	10.0
*n* = 17	1st Qu.	36.0	19.0	8.0	44.0	11.0
	**Median**	**37.0**	**22.0**	**10.0**	**46.2**	**12.0**
	Mean	38.4	21.3	10.4	45.3	12.2
	3rd Qu.	42.0	24.0	12.0	48.9	13.0
	Max.	45.0	27.0	16.0	51.3	14.0
C2.4	Min.	21.0	22.0	9.0	35.4	8.5
*n* = 11	1st Qu.	28.5	23.0	11.0	40.0	10.5
	**Median**	**30.0**	**24.0**	**14.0**	**41.6**	**11.0**
	Mean	30.5	24.6	15.2	42.3	11.2
	3rd Qu.	32.5	26.0	20.0	44.4	12.5
	Max.	38.0	27.0	21.0	49.7	14.0
C2.5	Min.	22.0	11.0	6.0	27.9	12.0
*n* = 5	1st Qu.	24.0	16.0	6.0	32.5	13.0
	**Median**	**26.0**	**16.0**	**6.0**	**33.4**	**14.0**
	Mean	27.2	17.0	6.8	33.1	13.6
	3rd Qu.	31.0	21.0	8.0	34.4	14.0
	Max.	33.0	21.0	8.0	37.5	15.0

1st Qu., first quarter; 3rd Qu., third quarter; ADL, ABC Dementia Scale (ABC-DS) score for activities of daily living (Domain A); BPSD, ABC-DS score for behavioral and psychological symptoms of dementia (Domain B); CDR-SOB, Clinical Dementia Rating sum of boxes; CF, ABC-DS score for cognitive function (Domain C); Max., maximum; Min., minimum; TDD, ABC-DS score for three-dimensional distance.

**Table 4:** ABC-DS and CDR-SOB scores for individuals with a baseline CDR score of 3

Group		ADL	BPSD	CF	TDD	CDR-SOB
C3.1	Min.	23.0	9.0	4.0	27.2	15.0
n = 10	1st Qu.	26.0	12.5	6.0	32.2	15.3
	**Median**	**31.0**	**15.0**	**9.0**	**36.7**	**17.0**
	Mean	31.4	15.0	8.4	36.2	16.4
	3rd Qu.	35.5	17.3	10.0	38.9	17.0
	Max.	42.0	22.0	14.0	46.2	18.0
C3.2	Min.	12.0	18.0	4.0	22.0	14.0
n = 17	1st Qu.	16.0	23.0	4.0	30.6	16.0
	**Median**	**24.0**	**25.0**	**8.0**	**34.2**	**18.0**
	Mean	21.9	23.9	9.1	34.4	16.9
	3rd Qu.	27.0	25.0	10.0	37.7	18.0
	Max.	37.0	27.0	22.0	49.8	18.0
C3.3	Min.	8.0	3.0	4.0	10.4	17.0
n = 6	1st Qu.	8.3	7.5	4.5	13.6	18.0
	**Median**	**10.5**	**9.0**	**6.0**	**15.1**	**18.0**
	Mean	10.8	8.8	5.3	15.3	17.8
	3rd Qu.	13.5	11.3	6.0	18.1	18.0
	Max.	14.0	13.0	6.0	18.9	18.0

1st Qu., first quarter; 3^rd^ Qu., third quarter; ADL, ABC Dementia Scale (ABC-DS) score for activities of daily living (Domain A); BPSD, ABC-DS score for behavioral and psychological symptoms of dementia (Domain B); CDR-SOB, Clinical Dementia Rating sum of boxes; CF, ABC-DS score for cognitive function (Domain C); Max., maximum; Min., minimum; TDD, ABC-DS score for three-dimensional distance.

**Table 5:** Characteristics of subgroups in comparison with C1.1

Subclass	Group	Compared with C1.1
C1.2, C2.1, C2.2	GI	BPSD not evident, but deteriorated cognitive function.
C1.3	–	Decreased ADL and CF and deteriorated BPSD.
C1.4, C2.3	GII	Slightly deteriorated BPSD. Deteriorated ADL and CF.
C2.4	–	Deteriorated ADL and CF without deterioration of BPSD.
C.2.5, C3.1	GIII	Deteriorated ADL, BPSD, and CF.
C3.2	–	BPSD not evident, but deteriorated ADL and CF.
C3.3	–	End stage

We characterized each subgroup along with GI, GII, and GIII by comparing them with C1.1, which served as a reference. For GI, the BSPD score was almost unchanged, but CF scores decreased. For C1.3, the ADL, BPSD, and CF scores declined. The differences between C.1.1 and GII were characterized by decreases in ADL and CF scores, with a slight deterioration in BPSD. The BPSD levels of C2.4 and C3.2 were comparable to that of C1.1, but the ADL and CF scores were considerably lower. GIII represents the advanced stage of the disease with high severity and showed markedly lower ADL, BPSD, and CF scores when compared with C1.1. C3.3 represents the terminal stage.

## DISCUSSION

The TDD score of the ABC-DS detected the changes in scores over 12 weeks for individuals with a baseline CDR of 0 and 0.5, 1, and 2. However, analysis of the total score for all 13 items in the ABC-DS only indicated changes among individuals with a baseline CDR of 0/0.5. MMSE and CDR-SOB failed to detect differences when the baseline CDR was 0/0.5. Using the ABC-DS, we could precisely monitor the dynamic changes in ADL, BPSD, and CF for AD patients.

**Fig.2:**
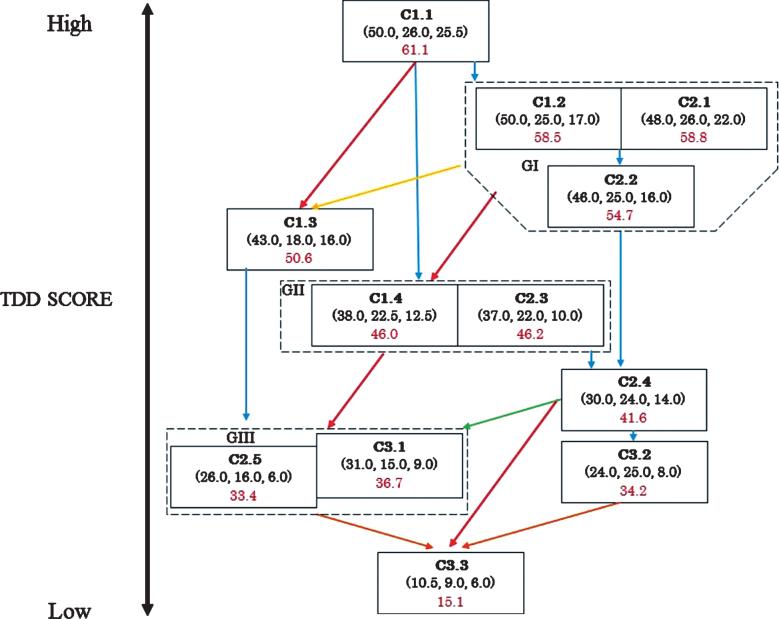
A hypothetical roadmap for Alzheimer’s disease progression. In each box, a class name (in bold), ADL score, BPSD score, CF score, respectively (in parentheses), and TDD score (in red font) are shown, from top to bottom, respectively. Blue line: Deterioration of ADL and/or CF score. Red line: Deterioration of ADL, BPSD, and CF score. Yellow line: Deterioration of BPSD score only. Green line: Deterioration of BPD and CF score. Orange line: Deterioration of ADL and BPSD score. GI: C1.2 and C2.1; GII: C1.4 and C2.3; GIII: C2.5 and C3.1.

Through cluster analyses, we classified 197 AD patients and found subgroups for patients with baseline CDR scores of 1, 2, and 3. Because these subgroups probably captured stages in progression, we hypothetically constructed the progression pathways by reasonably placing these subgroups in a roadmap, as shown in Fig. 2. We assumed that ADL, CF, and TDD scores tend to decrease or deteriorate as time passes. We configured the subgroups from top to bottom in Fig. 2, as falling the median of TDD scores shown as red numbers. We included colored arrow indicators showing probable potential directions for the patients regarding disease progression, to allow for the fact that the probability of a patient’s course following that of any pathway can differ depending on the patient’s condition.

To interpret the roadmap, we classified the arrow indicators with the following three kinds: blue, which indicates changes without deterioration in BPSD; red, which indicates deterioration of ADL, BPSD, and CF; and others, which mainly show a worsening of BPSD.

We interpreted the roadmap as follows. Patients moving from C1.1 to GI do not have BPSD. Patients at GI mainly take one of two pathways in terms of progression: GI to C2.4, in which ADL and CF deteriorate, but BPSD is not present; and GI to GII, in which BPSD appears and ADL and CF levels decrease. There is also a possibility to progress from GI to C1.3. Patients who move from C1.1 to C1.3 or GI to C1.3 could have BPSD at the early stage of progression. Patients who move from GI to C2.4 and C.3.2 could fail to show deterioration in BPSD to a great extent in their clinical course; however, patients at C2.4 may progress to GIII, showing apparent deterioration in BPSD. The patients in GII progress to GIII through a decline in ADL, BPSD, and CF. Patients from C1.3 to GIII show a decrease in ADL and CF levels. We define C3.3 as the terminal stage, which is dominated by patients from GIII. BPSD may not be evident in patients at C2.4 and C3.2, and some patients may take a pathway to the terminal stage, but the others at C2.4 or C3.2 could remain in the present subgroups.

We searched for relevant literature in PubMed to interpret the pathways in the road map as follows. Feldman reported that an MMSE score of 16 appears to be a turning point at which most instrumental ADL was lost, and the significant losses in basic ADL began to occur over the following 12 months [24]. As an MMSE score of 16 is equivalent to a TDD score of 49.5, this turning point was likely to be present at C1.3. Feldman’s observation can support our roadmap, indicating that patients at C1.3 moved to GIII through decreasing ADL levels.

Liu-Seifert et al. suggested that functional impairment is primarily driven by and follows the cognitive decline in mild AD dementia [21]. They likely observed the changes shown by the blue arrows in Fig. 2, and if so, the patterns of the changes they observed probably varied considerably, which is indicated by the many blue lines in Fig. 2.

Finally, Barocco et al. failed to prove, through an examination over a four-year time interval, the existence of the so-called “fast decliners” regarding AD progression [35]. Notably, the sample size of their study was small. As the changes from C1.1 to C1.3 or GII could be representative of fast decliners, we need further research on this issue.

Our study had limitations because our results of cluster analyses were preliminary and cross-sectional in nature; they represented only a static snapshot of the dynamically changing AD symptoms. The frequency of the paths (i.e., the arrows in Fig. 2) followed could vary considerably. As the rates of disease progression differ among AD patients, changes in the patterns of symptoms can be unique among patients. We, therefore, need to develop a roadmap by prospective observation studies so that we know how our hypothetical roadmap can reflect the actual progression pathways.

### Conclusion

The ABC-DS classified AD patients into subgroups characterized by ADL, BPSD, and CF states. In a previous study, we reported that the ABC-DS also identified subgroups in individuals considered to have probable MCI. Accordingly, we have determined that the ABC-DS can precisely monitor dynamic changes in the symptoms of individuals with conditions ranging from probable MCI to severe AD. To build upon our findings, we must confirm the roadmap using longitudinal data, showing changes from probable MCI to severe AD.

For the accurate estimation of overall severities, TDD is better than total scores because the total score neglects the fact the ABC-DS is composed of three dimensions.

## Supplementary Material

Supplementary Material
