# Supplementary material for: ABC Dementia Scale Classifies Alzheimer’s Disease Patients into Subgroups Characterized by Activities of Daily Living, Behavioral and Psychological Symptoms of Dementia, and Cognitive Function
Source: J Alzheimers Dis. 2020 Jan 7;73(1):383–92. doi: 10.3233/JAD-190767 (PMC7029317; doi:10.3233/JAD-190767)
Supplement: Supplementary Material [file jad-73-jad190767-s001.docx]

**Supplementary Material**

**ABC Dementia Scale Classifies Alzheimer’s Disease Patients into Subgroups Characterized by Activities of Daily Living, Behavioral and Psychological Symptoms of Dementia, and Cognitive Function**

# load “dataset.csv” where ADL, BPSD, CF scores of ABC-DS and Global-CDR of 1, 2, 3 are stored. Missing values are shown as “NA”.

clusdat<- read.csv("dataset.csv", header=T, na.strings="NA")

# Extract data of patients with Global-CDR of 1, for example.

clustdatd<- subset(clusdat, CDRBGL==1)

# Execute a hieralchial cluster analysis

clust.d1<-dist(clustdat)

hw<- hclust(clust.d1, "ward.D2")

# Show the result as a dendrogram

plot(hw, xlab="Patients with CDR 1")

**Supplementary Table 1.** Items of the ABC-DS

| Item | Domain | Question |
| --- | --- | --- |
| Q1 | Activities of daily living | Daily activities: When the patient changes his/her clothes, how is he/she? |
| Q2 |  | Motivation: How willingly does the patient undertake activities of daily life? |
| Q3 |  | Communication: When the patient wants to communicate with others, how easily can he/she do it? |
| Q4 |  | Complex acts: When the patient wants to use electric appliances such as a TV or an air conditioner, how well can he/she do it? |
| Q5 | Cognitive function | Recent event memory: How well can the patient recall the place where he/she put his/her belongings? |
| Q6 |  | Recent event memory: How long can the patient remember daily happenings? |
| Q7 | Behavioral and psychological symptoms of dementia | Restlessness: When the patient is required to sit still, how is he/she? |
| Q8 |  | Irritability: When the patient feels something is unsatisfactory, how is he/she? |
| Q9 |  | Cooperativeness: When caregivers ask the patient something, how is he/she? |
| Q10 | Cognitive function | Medication: When the patient takes medication, how much help does he/she need? |
| Q11 | Activities of daily living | Meals: When the patient takes meals, how is he/she? |
| Q12 |  | Toilets: When the patient uses the toilet, how much help does he/she need? |
| Q13 | Cognitive function | Care-burden: How frequently should caregivers see to the patient? |


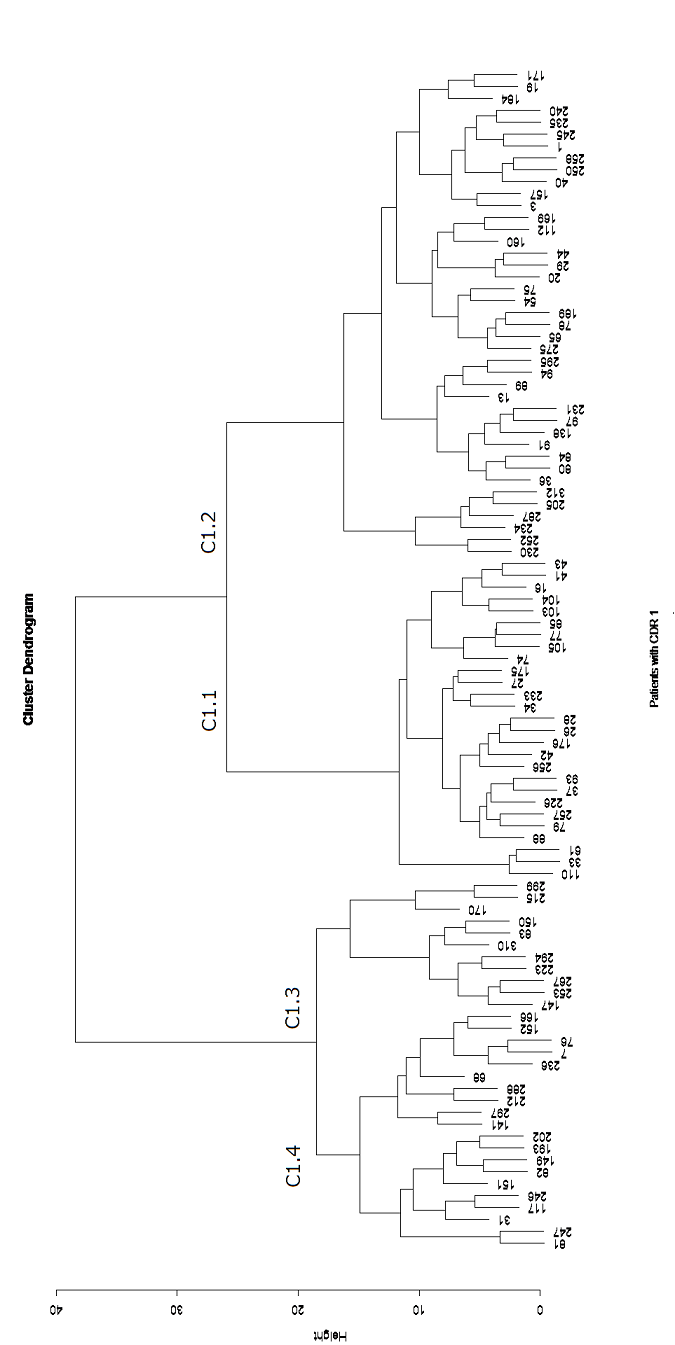


**Supplementary Figure 1**


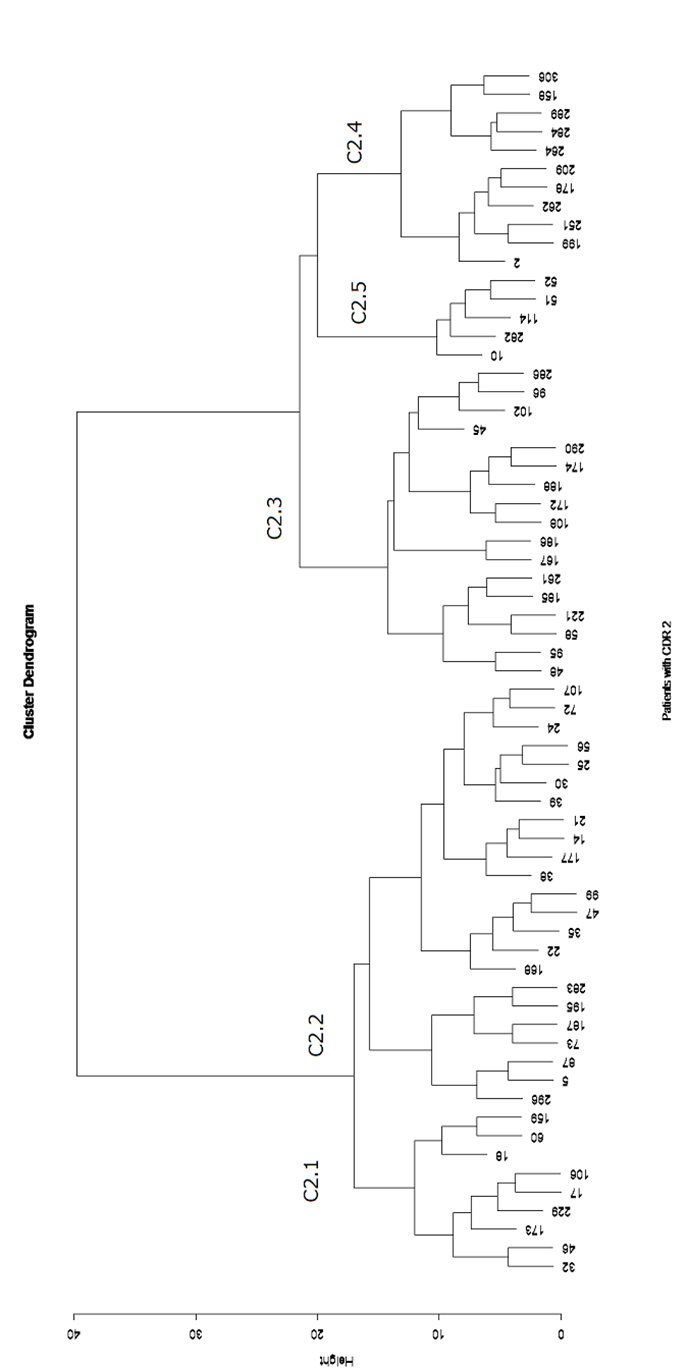


**Supplementary Figure 2**


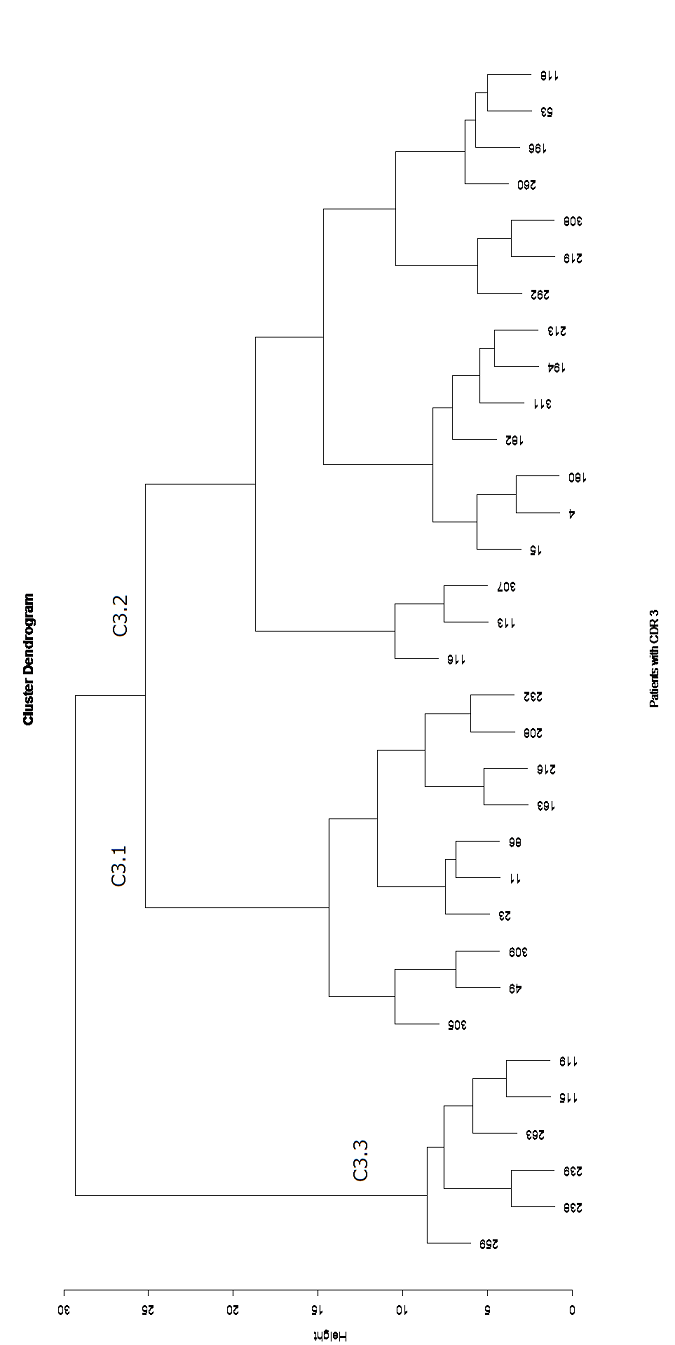


**Supplementary Figure 3**
